# Topical Ophthalmic Anesthetics for CornealAbrasions: Findings from a Cochrane SystematicReview and Meta-Analysis

**DOI:** 10.21203/rs.3.rs-4160700/v1

**Published:** 2024-06-04

**Authors:** Irene Kuo, Louis Leslie, Su-Hsun Liu

**Affiliations:** Wilmer Eye Institute Johns Hopkins University School of Medicine

**Keywords:** corneal injuries, corneal abrasion, eye pain, local anesthetics, corneal epithelium, emergencyroom visits

## Abstract

**Background:**

Despite potential benefit, outpatient use of topical ophthalmic anesthetics can result in poor healing, infection, scar, and blindness. An unbiased analysis of randomized controlled trials (RCTs) is needed to examine their effectiveness and safety compared with placebo or other treatments for corneal abrasions.

**Methods:**

Cochrane Central Register of Controlled Trials, MEDLINE, Embase.com, Latin American and Caribbean Health Sciences, ClinicalTrials.gov, and the World Health Organization International Clinical Trials Registry Platform were searched on February 10, 2023, without restriction on language or publication date.

**Results:**

Systematic review and meta-analysis of nine RCTs describing 314 participants with post-traumatic abrasions and 242 participants with post-surgical abrasions, with a median study length of 7 days (interquartile range, 7–14), show no evidence of a difference in pain control between anesthetics and placebo at 24 hours in post-trauma cases. Self-reported pain at 24 hours is reduced with anesthetics plus topical nonsteroid anti-inflammatory drug in post-surgical participants (mean difference [MD], −5.72 on a 10-point scale; 95% CI, −7.35 to −4.09; 1 RCT; 30 participants) and at 48 hours with anesthetics alone in post-trauma participants (MD, −5.68; 95% CI, −6.38 to −4.98; 1 RCT; 111 participants). Anesthetics are associated with 37% increased risk of non-healing defects (risk ratio, 1.37; 95% CI, 0.78 to 2.42; 3 RCTs; 221 post-trauma participants). All evidence is of very low certainty. Over 50% of trials have an overall high risk of bias.

**Conclusions:**

Available evidence is insufficient to support outpatient use of topical anesthetics for corneal abrasions with respect to pain, re-epithelialization, and complication risk.

## PLAIN LANGUAGE SUMMARY

The cornea, the clear, dome-shaped structure covering the iris, is one of the most pain-sensitive tissues in the body. Traumatic abrasions (disruption of the epithelium, the top corneal layer) account for > 10% of all eye-related visits to emergency rooms in the US. Epithelial removal also occurs in some eye surgeries. Topical anesthetic drops for corneal abrasions are controversial; they can be habit-forming and can cause corneal scar or blindness. Nonetheless, a growing number of emergency department providers prescribes these drops for corneal abrasions, despite the fact most heal within days. By carefully analyzing 9 randomized controlled trials, we do not find enough evidence that topical anesthetics reduce pain, improve healing, or decrease the chance of complication compared with placebo.

## INTRODUCTION

Corneal abrasions, loss of the corneal epithelium, can occur from trauma, ocular surgery, dryness, and exposure. They account for 13% of eye-related emergency department visits in the US, with an annual incidence of 3 per 1000 persons and a higher male prevalence.^1–3^ Traumatic abrasions present with symptoms like pain, photophobia, redness, and tearing. As the cornea is one of the most highly innervated tissues in the body, corneal nociceptors, especially mechanoreceptors, contribute to intense eye pain until healing occurs. Although most small to medium-sized corneal abrasions heal within a few days, complications like corneal erosions and infection may require medical or surgical intervention. In contrast, post-surgical corneal epithelial defects benefit from a sterile field and controlled wound construction.

In medicine, topical nonsteroidal anti-inflammatory drugs (NSAIDs) and anesthetic drugs are used for localized analgesia with limited systemic effects. However, numerous case reports describe ophthalmic complications from use of high concentrations of topical anesthetics, at excessive frequency, or for prolonged periods of time, including corneal infiltrate, stromal keratolysis (“melt”), endothelial injury, and perforation^4–21^ – all of which can lead to vision loss. Recent emergency department studies have deemed outpatient use of prescribed topical anesthetics as safe and effective for corneal abrasions.^22–24^ This assessment contrasts with the experience of ophthalmologists,^6–23^ leading to renewed questions about outpatient anesthetic use. The lack of consensus guidelines may account for variations in practice within and across specialties.^25–27^

The main objective of this summary of our Cochrane systematic review is to report the comparative effectiveness and safety of topical ophthalmic anesthetics compared with placebo or other treatments for corneal abrasions of any etiology, based on the best available evidence.^28^

In nine eligible randomized controlled trials (RCTs) describing 556 participants with corneal abrasions from traumatic or surgical origin, we find insufficient evidence to support outpatient use of topical ophthalmic anesthetics compared with placebo for reducing pain, improving re-epithelialization, or reducing complication risk in corneal abrasions.

## METHODS

### Eligibility criteria for studies and outcomes of interest:

We examined only randomized controlled trials (RCTs). Eligible trials compare topical ophthalmic anesthetics (amide or ester class) with a control group (placebo, non-treatment, or alternative treatment). Trials in which topical anesthetics with an NSAID were compared with a control group were eligible. We considered trials that enrolled participants of all ages with corneal abrasions within 48 hours of presentation from accidental trauma or ophthalmic surgery.

The primary review outcomes were (1) mean reduction in self-reported pain on a visual analog scale (VAS), where smaller numbers represent less pain, at 24, 48, and 72 hours after treatment initiation; (2) the proportion of participants without complete resolution of epithelial defects by 24–72 hours; and 3) the proportion of participants with adverse events (e.g. microbial keratitis or stromal infiltration, corneal stromal thinning, corneal perforation, surgical interventions) reported at the longest follow-up time of the study. For secondary outcomes, we considered treatment failure (the proportion of participants who required rescue oral analgesics by 72 hours after treatment initiation) and quality of life assessed by a validated instrument at the longest follow-up time of the study. Trials were not excluded on the basis of outcome reporting.

### Search methods for identifying studies:

We searched the Cochrane Central Register of Controlled Trials (CENTRAL), MEDLINE Ovid, Embase.com, ClinicalTrials.gov (www.clinicaltrials.gov), and the WHO International Clinical Trials Registry Platform (ICTRP) (www.who.int/ictrp/search/en) to identify potentially eligible RCTs for this review. We did not impose restrictions on the search date or language of publication. The last date of search was February 10, 2023.

#### Study selection:

Pairs of review authors worked independently to review titles and abstracts as well as full-text records against the eligibility criteria; disagreements were resolved by discussion.

#### Data collection and risk of bias:

Authors independently extracted the following data from included studies: trial characteristics, methods, participants, interventions, outcomes, and source of funding. Two review authors independently applied the Cochrane's Risk of Bias version 2 (RoB2) tool^29^ to assess risk of bias in the efficacy outcome (pain control by 48 hours) and the safety outcome (complications at the longest follow-up time). We judged each study that reported either outcome to have been at “low risk,” “high risk” or raising some concerns for risk of bias. An overall Grading of Recommendations, Assessment, Development, and Evaluations (GRADE) assessment of the certainty level of evidence was performed based on four main criteria: risk of bias, inconsistency, indirectness, and imprecision.^30^ Disagreements were resolved by discussion within the author team.

#### Data analysis and synthesis:

For the continuous outcome of pain assessment, we estimated the difference in means (“mean difference”) (MD) with 95% confidence intervals (CI). For dichotomous outcomes, we estimated the risk ratios (RR) with 95% CI. We estimated the risk difference (RD) with 95% CI for trials reporting no events in either treatment group.^31^

To determine whether results could be combined in meta-analyses, we assessed the trials for clinical and methodological heterogeneity in trial design, eligibility of trial participants, intervention and comparator differences, and outcome definitions.^32,33^ We evaluated the amount of statistical heterogeneity using the I^2^ statistic outlined in the Cochrane Handbook.^33^

## RESULTS

**Figure 1:**
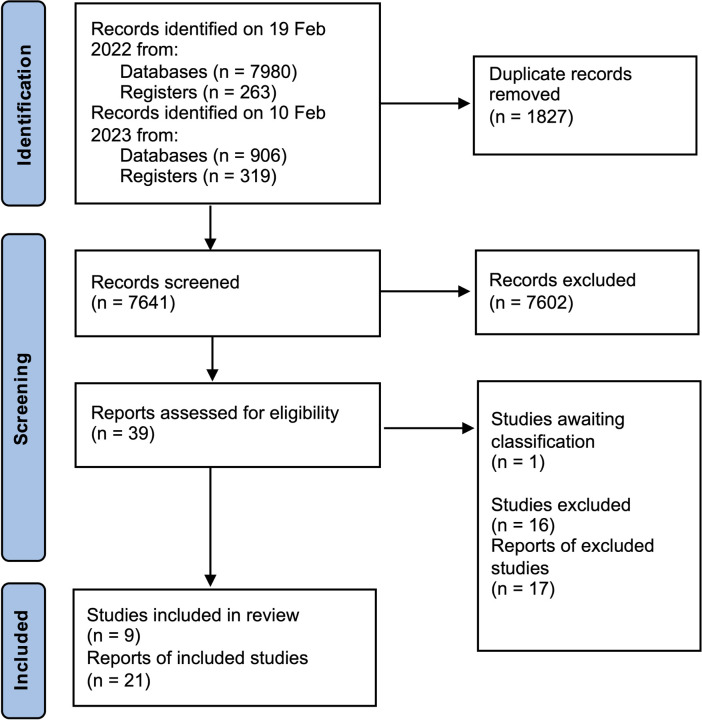
Preferred Reporting Items for Systematic Reviews and Meta-Analysis (PRISMA) flow diagram showing identification and selection of randomized controlled trials that compare different topical anesthetics with or without co-interventions to placebo or active control for corneal abrasions.

We present analysis of nine RCTs (21 study reports) after a literature search yielded 7641 unique titles and abstracts that were screened, of which we reviewed 39 potentially relevant full-text publications (Fig. 1). Our quantitative analysis includes 8 of the 9 RCTs. One trial does not report data in a manner amenable to analysis for our specified outcomes.^34^

All included trials are parallel-group, 2-arm RCTs, except for one 7-arm trial.^35^ The trials span across eight countries from 1994 to 2021. The four post-traumatic abrasion trials were conducted in emergency departments. Four trials did not receive industry funding; funding information is unknown for five trials.^34–38^

The included trials report data from 626 eligible participants who were randomized. After selecting the appropriate 3 arms from a 7-arm trial,^35^ we analyzed 556 participants with a median of 45 participants per trial (interquartile range [IQR], 44–74; 9 RCTs). A higher proportion of women are enrolled in surgical trials (60%; 166/278 participants; 4 RCTs) than in post-trauma settings (21%; 65/314 participants; 4 RCTs).

The following topical anesthetics are compared with placebo (Table 1): tetracaine 1% (2 RCTs),^23,39^ 0.5% (1 RCT),^22^ and 0.4% (1 RCT);^40^ proparacaine 0.05% (3 RCTs);^34,35,38^ and lidocaine 2% (1 RCT).^36^ One multi-arm trial compares proparacaine 0.05% and proparacaine 0.05% with topical diclofenac 0.1% against placebo. ^35^ One trial has an NSAID active control (diclofenac 0.1%).^37^ When reported, the total amount of topical anesthetic dispensed by emergency departments ranges from 1.5 mL to 40 mL (approximately 30–800 drops).

Four studies analyze abrasions of traumatic etiology in 314 participants (314 eyes).^22,23,38,40^ Most abrasions involve corneal foreign bodies (47%; 148 eyes) or direct trauma (19%; 61 eyes). Five studies describe 242 participants (256 eyes) with post-surgical corneal defects, four from photorefractive keratectomy (PRK)^34,35,37,39^ and one from pterygium surgery.^36^ Overall, the median study length is 7 days (IQR, 7–14 days) with a median of 11 days (IQR, 7–18 days) for post-trauma trials and 7 days (IQR, 3–7 days) for post-surgical trials. Overall, the median treatment duration is 24 hours (IQR, 24–168 hours) with a median of 36 hours (IQR, 24–78 hours) for post-trauma trials and 24 hours (IQR, 24–168 hours) for post-surgical trials.

Two (67%) of the three trials describing ocular pain results have an overall high risk of bias. Five (71%) of the seven trials describing ocular complications have an overall high risk of bias, primarily from missing outcome data and selective reporting of results.

### Effectiveness and safety of interventions

#### Critical outcomes

##### Pain control from baseline to 24 hours after treatment initiation

**Figure 2:**
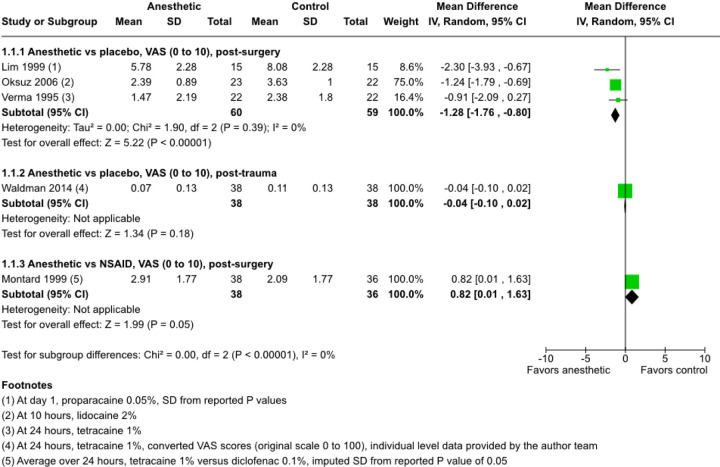
Forest plot showing mean differences with their 95% confidence intervals (CI) for self-reported pain at 24 hours. Compared with placebo, topical anesthetics are associated with a moderate reduction in self-reported pain at 24 hours in post-surgical trials but show little or no difference in effect in post-trauma participants. However, compared with NSAIDs in post-surgical participants, self-reported pain is increased in the topical anesthetic group compared with the NSAID group. CI = confidence interval; IV = Inverse Variance; SD = standard deviation; VAS = visual analog scale.

The combined estimate for pain scores reported by post-surgical participants^35,36,39^ suggests that when compared with placebo, topical anesthetics reduce pain by 1.28 on a 10-point VAS (MD, −1.28; 95% CI, −1.76 to −0.80; 3 RCTs; 119 participants; Fig. 2). In contrast, one post-PRK trial reports 0.82 higher pain scores on a 10-point VAS with tetracaine compared with NSAID (MD, 0.82; 95% CI, 0.01 to 1.63; 74 participants; Fig. 2).^37^

No evidence of a difference in pain control (MD, −0.04; 95% CI, −0.10 to 0.02) is found in a trial using a mixed-model to account for multiple measurements, to which 76 (62%) of 122 of post-trauma participants contributed data.^23^

One post-surgical trial compares an anesthetic plus an NSAID with placebo.^35^ The estimated difference in means in reduction in pain scores (i.e., the “mean difference” or MD) from this study indicates anesthetic plus NSAID reduces pain scores at 24 hours when compared with placebo (MD, −5.72 on a 10-point scale; 95% CI, −7.35–−4.09; 30 participants).

The certainty of evidence for all effect estimates is very low because of potential risk of bias and imprecision.

##### Pain control from baseline to 48 hours after treatment initiation

Using a 10-point VAS, post-surgical participants of one trial report little pain control relative to placebo (MD, 0.41; 95% CI, −0.45 to 1.27; 44 participants.^39^ Post-trauma participants in one trial report 5.68 lower pain scores with tetracaine 0.5% than placebo (MD, −5.68; 95% CI, −6.38 to −4.98; 111 participants).^22^ One post-trauma trial is excluded from this analysis because 57% of 124 randomized participants are lost to follow up.^23^ The overall certainty of evidence is very low because of a high risk of bias, imprecision, and inconsistency across studies.

No trial records pain control from baseline to 72 hours after treatment initiation.

##### Epithelial healing by 24 to 72 hours

**Figure 3:**
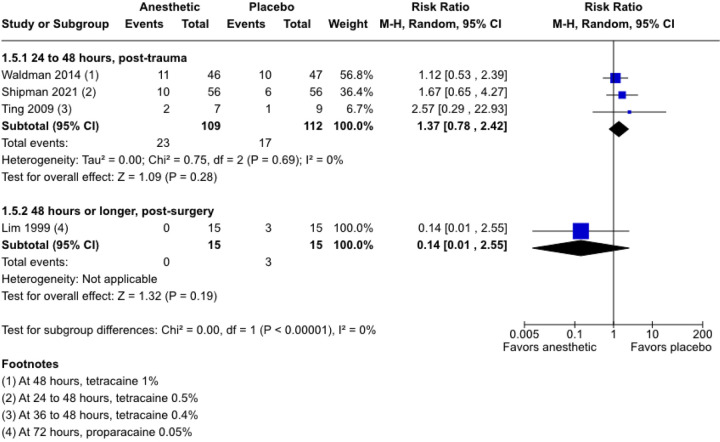
Forest plot showing estimated risk ratios (RR) with their 95% CI, for proportions of participants with persistent epithelial defects. The proportion of post-trauma participants with persistent epithelial defects is higher with topical anesthetics than with placebo or NSAID. However, in post-surgical participants, the proportion of unresolved epithelial defects is higher with placebo than with topical anesthetics. CI = confidence interval; M-H = Mantel-Haenszel.

In three post-trauma trials^22,23,40^ the risk of persistent epithelial defects assessed at 24 to 72 hours is 37% higher with varying concentrations of tetracaine compared with placebo (RR, 1.37; 95% CI, 0.78 to 2.42; 3 RCTs; 221 participants; I^2^ = 0%; Fig. 3). However, a decrease in the proportion of participants with persistent defects is found in a trial of 30 post-surgical participants using proparacaine 0.05% (RR, 0.14; 95% CI, 0.01 to 2.55; Fig. 3) and proparacaine 0.05% plus diclofenac 0.1% (RR, 0.33; 95% CI, 0.04 to 2.85), compared with placebo.^35^

Overall, there is no evidence of differences although the evidence is of very low certainty because of a high risk of bias, inconsistency, and imprecision.

### Complications reported as having occurred by the longest follow-up time

**Figure 4:**
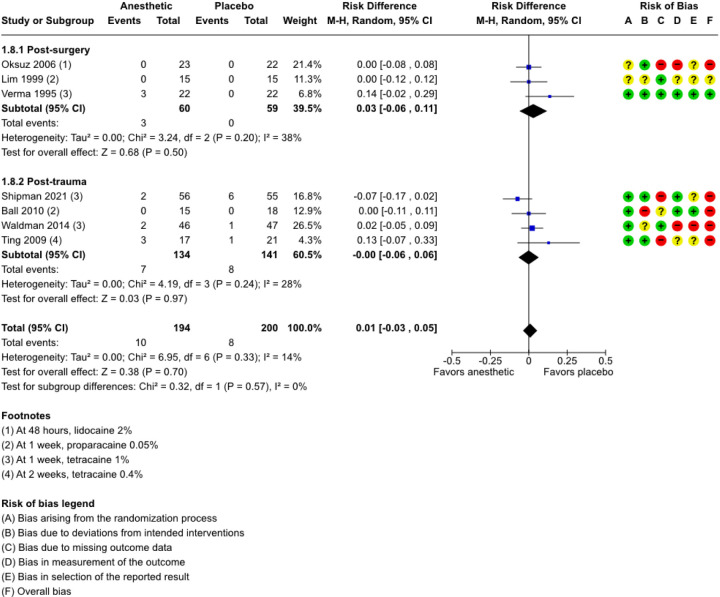
Forest plot showing estimated risk difference with 95% CI, for complications. There is no evidence of a difference in absolute risk with topical anesthetic use versus placebo or NSAID. CI = confidence interval; M-H = Mantel-Haenszel.

Seven trials report the proportion of individuals with this outcome.^22,23,35–39^ The longest follow-up time is two weeks; the shortest is 48 hours.^36^ Three of the seven trials report no events of complication in either trial arm.^35,36,38^ In the comparison of topical anesthetics versus placebo or NSAID, topical anesthetics are associated with a higher proportion of complications in post-trauma participants at up to two weeks (RR, 1.13; 95% CI, 0.23 to 5.46; 3 RCTs; 242 participants; supplemental figure). For post-surgery participants at up to one week, the risk of complications also is higher with topical anesthetics (RR, 7.00; 95% CI, 0.38 to 128.02; 1 RCT; 44 participants; supplemental figure).^39^ We also estimate risk differences (RDs) and find no evidence of differences in absolute risks for complications up to one week in both the post-surgery trials (RD, 0.03; 95% CI, −0.06 to 0.11; 3 RCTs; 119 participants; Fig. 4)^35–37^ and post-trauma trials (RD, 0.00; 95% CI, −0.06 to 0.06; 4 RCTs; 275 participants; Fig. 4).^22,23,38,40^ In one post-trauma trial, patients’ self-reported pain level is collected by telephone interview at the two-week mark.^40^

In the single study comparing anesthetic plus NSAID versus placebo, proparacaine 0.05% plus diclofenac 0.1% versus placebo, the authors report no adverse events in either treatment arm up to one week post-surgery (RD, 0.00; 95% CI, −0.12 to 0.12; 30 participants).^35^

For both comparisons, the certainty of evidence is very low because of potential risk of bias and imprecision.

### Important outcomes

In the one study that reports treatment failure by 72 hours after treatment initiation, no oral analgesic use is used in either arm from 24 hours to 2 weeks.^40^ The evidence of a difference between topical anesthetics and placebo is of very low certainty because of a potential risk of bias and imprecision. No study assesses quality of life.

## DISCUSSION

Corneal abrasions from trauma or epithelial defects created during ophthalmic surgery occur commonly. In this review of nine RCTs in which a total of 556 participants with corneal abrasions are analyzed, we compare the safety and efficacy of topical ophthalmic anesthetic with or without NSAID versus placebo or NSAID. The evidence is of very low certainty for any difference between topical anesthetic and placebo in pain control, healing, or outcomes suggestive of anesthetic abuse. The main reasons for this level of certainty are small sample sizes and an overall high risk of bias. Use of anesthetic eye drops without close monitoring or attention to the amount dispensed (in one trial, 40 mL was prescribed for outpatient use)^38^ creates a potential for significant ocular complications. Such sequelae may not be observed in trials with short follow-up or with high participant drop-out, characteristics of several trials in this review.

Given the number of abrasions evaluated in emergency departments (EDs) each year, investigators should plan for larger sample sizes and a longer duration of follow-up, perhaps having a follow-up ophthalmic examination to confirm lack of recurrent erosion or contact lens-related keratitis.^1,41,42^ These two conditions also present with disruption of the corneal epithelium but require ophthalmic care. Because most abrasions heal within a few days with just a topical antibiotic, high participant attrition in any such trial is anticipated, yet the dangers of anesthetic use increase with lack of monitoring, longer duration of use, higher concentration, or more frequent dosing than prescribed.^43,44^ For a self-limited condition, the risk of blindness from corneal melt, infection, or perforation—all findings of topical anesthetic abuse—must be weighed against any possible benefit.

The current review cannot address whether the numerous case reports of anesthetic complications describe outliers since most trials do not assess safety outcomes beyond one to two weeks. The case literature suggests certain patient populations may be at risk for topical anesthetic abuse.^4–21^ These characteristics include a history of psychiatric illness, depression, dry eye, or drug abuse; certain occupations like manual labor that might prioritize return to work over close follow-up or recovery; or a medical or veterinary background that provides access to topical anesthetics. There are ethical concerns with enrolling participants at higher risk for anesthetic abuse.

In any future trial, sample size assumptions (effect size, power) should be justified, and efficacy outcomes should include a more complete understanding and documentation of pain. Baseline and follow-up pain levels, function, and quality of life as well as complete demographic data would aid interpretation of applicability. Some trials record the baseline pain level in participants; others do not. A high proportion of participant drop-out is noted in one trial of post-traumatic abrasions.^23^ This same trial employs a mixed-model approach for the multiple, repeated self-reported pain measurements from individuals that are “adjusted for pain on arrival, and allowing for individuals with different pain levels and pain reducing at different rates.”^23^ No other included trial utilizes this method. Additionally, reliance on self-reported pain intensity following surgery can result in an overestimation of the needed intervention.^45,46^ Lessons gained from the current opioid epidemic include the need for better understanding of pain, use of other treatment modalities, and focus on returning patients to normal function.^45,46^ Given that topical anesthetic use also can be habit-forming, the same considerations may apply to pain control in corneal abrasion.

A smaller systematic review of topical anesthetics for traumatic abrasions that includes 2 trials in this current review similarly concludes that topical anesthetic use is “currently not supported by evidence.”^47^ One summary (not a systematic review) of interventions for post-PRK pain recommends against the long-term use of topical anesthetics.^48^ Authors of another summary (not a systematic review) state topical anesthetics do not delay epithelial healing.^49^ A third highlights the need for careful monitoring.^50^ The small review^47^ excludes a trial we had included,^34^ and all three exclude two non-English language trials that we had included.^35,37^ The American Academy of Ophthalmology’s 2022 Refractive Surgery Preferred Practice Pattern® states “small quantities of dilute topical anesthetic are sometimes used but warrant close supervision.”^51^ Treatment guidelines and point-of-care resources generally cite case reports and series when recommending against the use of topical anesthetics for pain control.^43,44,52^ In the United Kingdom, authors of the Clinical Knowledge Summary by National Institute for Health and Care Excellence (NICE) state that topical anesthetic can be used to aid examination but “repeat doses should be avoided as they can cause corneal epithelium toxicity and impair healing.”^53^

A few years ago, the American Academy of Ophthalmology invited the American College of Emergency Physicians to issue joint guidelines regarding use of topical anesthetics for corneal abrasions because of concerns of potential harm. A joint working group from both organizations convened and reviewed the peer-reviewed literature. However, sidestepping concerns of the ophthalmologists regarding clinical recommendations, the emergency physicians of the group recently published guidelines without any ophthalmologist authors.^27^ The guidelines endorse dispensing 1.5 to 2 mL topical anesthetics (q30 minutes for 24 hours) for corneal abrasions in the emergency department setting.^27^ This amount (60–80 drops) actually would last 30–40 hours, although the guidelines state patients would be asked to discard drops after 24 hours. Guideline authors question the emphasis our Cochrane Systematic Review places on RCTs, question its relevance because it is “written solely” by ophthalmologists (not true), and claim an “existing practice pattern of ophthalmologists prescribing topical anesthetics for photorefractive keratectomy postoperative analgesia either for initial use or for breakthrough pain” (not accurate). Topical anesthetic use is exceedingly rare after PRK, and at least three papers the authors cite to support their use ironically describe complications or increased postoperative pain from anesthetic use.

Only four trials in the current review report specific adverse events; most do not report events beyond one week. This short follow-up may lead to an inaccurately low estimate of adverse event rates. Topical anesthetic abuse is usually described in the literature at time points well beyond those of the trials in this review. In Turkey, where topical anesthetics were sold over the counter until 2012, one study found that median duration of use was 28 days (range, 10–112 days; 7 patients) before amniotic membrane transplantation was needed for corneal melt.^54^ In a case series of patients with toxic keratopathy from topical anesthetic abuse, epithelial healing took a median of 17 days (range, 6–50 days; 19 patients).^21^

The time from trauma to participant randomization is longer than for surgical participants by 24 to 36 hours. Post-trauma trials vary in whether patients with rust rings are included, which has implications for potential risk of bias and applicability of findings. Abrasion size in post-trauma trials likely varies as well, whereas the epithelial defect is more uniformly created in surgical procedures. Therefore, the baseline risk for slow epithelial healing and adverse events such as infection may differ systematically by setting. Because of different baseline risks, combining post-trauma and post-surgical subgroups when examining for complications does not make clinical sense and therefore is not done.

In conclusion, there is insufficient evidence that topical anesthetics are more safe or effective than placebo for pain or healing of corneal abrasions of either surgical or traumatic origin. Trials with larger sample sizes, means to minimize attrition of trial participants, efficacy outcomes that reflect a better understanding and measurement of pain, and differentiation of abrasions (e.g., uncomplicated abrasions vs. ones with retained foreign material [or “rust rings”]) would reduce bias and increase certainty of evidence. Future trials and systematic reviews should include core outcome sets, comprised of multistakeholder consensus of measurable, reported standardized outcomes to improve consistency across studies and facilitate comparison of interventions.^55–59^ Given that most abrasions are self-limited, trial participants should receive appropriate quantities of topical anesthetic to reduce the risk of complication and vision loss. Lessons learned from the opioid epidemic can apply to analgesia for corneal abrasion.

## Supplementary Material

Supplement 1

## Data Availability

This article is based on a published Cochrane Review. The data and data use policy can be found in the Cochrane Database of Systematic Reviews (CDSR) (see www.cochranelibrary.com for information). Cochrane Reviews are updated as new evidence emerges and in response to feedback; the CDSR should be consulted for the most recent version of the review.
