# Supplementary material for: Topical Ophthalmic Anesthetics for CornealAbrasions: Findings from a Cochrane SystematicReview and Meta-Analysis
Source: Res Sq. 2024 Jun 4:rs.3.rs-4160700. Preprint. [Version 1] doi: 10.21203/rs.3.rs-4160700/v1 (PMC11177972; doi:10.21203/rs.3.rs-4160700/v1)
Supplement: Supplement 1 [file NIHPPrs4160700v1-supplement-1.pdf]

Supplementary Files

This is a list of supplementary files associated with this preprint. Click to download.

- SuppFig1.pdf
